# Thyroid cytology: practical tricks and pitfalls

**DOI:** 10.1007/s00428-025-04349-2

**Published:** 2025-11-22

**Authors:** Esther Diana Rossi, Alessia Piermattei, Federica Cianfrini, Natalia Cappoli, Antonino Mulè, Liron Pantanowitz

**Affiliations:** 1 Division of Anatomic Pathology and Histology-Fondazione, Policlinico Universitario “Agostino Gemelli”-IRCCS, Università Cattolica del Sacro Cuore, Largo Francesco Vito, 1, 00168 Rome, Italy; 2 Department of Pathology, University of Pittsburgh, Pittsburgh, USA

**Keywords:** Fine needle aspiration cytology, Indeterminate lesions, Thyroid malignancies, Immunocytochemistry, Personalized medicine

## Abstract

Thyroid lesions are a common finding, especially in the adult population, based on the evidence that more than 50% of individuals have thyroid nodules. The increasing detection of these lesions is mostly due to frequent ultrasonographic head and neck evaluation, which can now identify small subcentimeter nodules. Fortunately, most of these nodules are benign (70%), with only 5–10% of them attributed as malignant lesions. However, the remaining 20% falling into the category of indeterminate lesions which can lead to some pitfalls and tricky evaluations. Since 1996, different classification systems have been introduced and among them, the most worldwide adopted is the Bethesda System for Reporting Thyroid Cytopathology (TBSRTC). It is well-known that TBSRTC as well as other classification systems, subclassified indeterminate lesions into subgroups which specifically for the TBSRTC include a) atypia of undetermined significance (AUS), b) follicular or oncocytic cell neoplasm (FN) and c) suspicious for malignancy (SFM). However, despite the high positive predictive value (97%-99%), sensitivity (65%-99%) and specificity (72%-100%) of thyroid FNAC, diagnostic pitfalls exist that can lead to false positive and/or false negative results. This inconvenience is mostly due to the overlapping of morphological features in terms of cells and even background. This review discusses the most important potential pitfalls in the cytologic evaluation of thyroid lesions that can lead to such diagnostic errors.

## Introduction

Thyroid nodules are frequently encountered in clinical practice, reflecting their prevalence in the general population, especially among adults and mostly women [1–6]. Fine needle aspiration cytology (FNAC) combined with ultrasound (US) analysis of the thyroid gland are the first and most valuable diagnostic tools for the pre-surgical evaluation of thyroid lesions, leading to a conclusive cytologic diagnosis in up to 80% of cases [1–6]. An extensive review of the literature highlighted that many thyroid nodules (70–75%) are benign, with only a small subset of malignant nodules that need to be pre-surgically identified for tailored management [1–3].

Furthermore, the adoption of specific terminologies, introduced by different thyroid reporting systems, has helped effectively communicate thyroid FNAC diagnoses in a clear and understandable way [4–13]. In these reporting systems, cytomorphology is typically combined with an expected risk of malignancy (ROM) and coupled to clinical and/or surgical patient management [4–13]. The purpose of any classification system is to clarify and tailor patient management, and minimize inter-and intra-observer agreement. Since 1996, when the Papanicolaou Society thyroid cytological classification was introduced, there had been several other systems, with also revisions and further editions of them including in 2005 the first Japanese and then in 2007 the Bethesda System for Reporting Thyroid Cytopathology (TBSRTC), which subsequently underwent two revisions [8–10]. Other international thyroid terminology classifications include the British, Italian, Australasian and other Japanese cytology systems [4–13].

Among the others, TBSRTC has significantly changed the approach to thyroid cytology due to the worldwide spread and acceptance of it leading also to be endorsed in 2015 by the American Thyroid Association (ATA) thyroid cancer guidelines as a valid system to diagnose thyroid cytology [14]. TBSRTC has also been recognized in the 4th and 5th editions of the WHO classification of Endocrine and Neuroendocrine Tumors [15–17]. Furthermore, different cytologic societies, such as the International Academy of Cytology (IAC) and the European Federation of Cytology Societies (EFCS) have supported and endorsed the third edition of the Bethesda, confirming the leading role of this classification system worldwide [7, 8].

Recently, in July 2023, TBSRTC published a 3rd edition which included the refinement of diagnostic categories such as subdivisions for Atypia of Undetermined Significance (AUS), updated ROM’s including for the pediatric population, modified specific management strategies, adding a chapter about the ultrasound evaluation of thyroid nodules, and acknowledging the performance of molecular testing [8]. The important change made in the AUS category, with a subclassification into nuclear atypia and others [8], reduces differences in the indeterminate categories compared with the other international classification systems [10–13].

However, despite the adoption of a specific classification system, it is also well-known that thyroid FNAC yields a high positive predictive value for identifying malignancy, ranging from 97%-99% in to 95% according to TBSRTC and British thyroid system and the Italian classification system [9–12].

Nonetheless, despite the high diagnostic accuracy of FNAC, it is not uncommon to encounter false positive and/or false negative results due to a variety of quantitative and qualitative aspects of the aspirated material [1–3, 18–20]. Whilst false negative results are related mostly to issues of sample adequacy, false positive results are usually due to a broad range of causes including, among others, the fact that some entities exhibit cytomorphologic appearances that overlap with other tumor subtypes [20–24] (Tables 1, 2, and 3). For an example it is not uncommon to encounter nuclear pseudo-inclusions not only in papillary thyroid carcinoma (PTC), but also focally in medullary thyroid carcinoma (MTC), Anaplastic thyroid carcinoma (ATC), metastatic melanoma, and even some benign entities such as hyalinizing trabecular tumor (HTT).

**Table 1:** Possible pitfalls for the diagnostic categories

Diagnoses	FN	FP
Non-Diagnostic (ND)	Few cells suggestive of benign condition	AUS
Cystic lesions	Cystic degeneration and squamous cells	Atypical cyst-lining cells, few cells with features suggestive for SFM or even PM
Grave’s disease (GD)	NONE	PTC, OFN
Lymphocytic thyroiditis (LT)	Scant diagnostic features of LT	Mostly PTC; Lymphomas; OFN
AUS	Scant cellular component	FN, PTC, SFM, PDTC
FN	Underestimate architectural and cellular features; Intrathyroid parathyroid adenoma; PTEN hamartoma	SFM/PM; PTC; FVPTC; FC
OFN	HT; Goiter, Granular cell tumor; Intrathyroid parathyroid adenoma;	PTC*; MTC; HT
NIFTP	Follicular-patterned lesions	SFM or PM favoring PTC; MTC
PTC	HTT; LT; GD	MTC; PDTC
MTC	SFN/FN; FNHCT	PTC, HTC
PDC	SFN/FN; FNHCT	MTC; Metastases; Lymphoproliferative disorders
ATC	Few atypical cells classified as ND	PDTC; MTC; Metastases; Lymphomas
METASTATIC LESIONS	Few atypical cells classified as ND	PDTC, MTC, ATC

*FN* false negative, *FP* false positive, *HT* hashimoto thyroiditis, *AUS* atypia of undetermined significance, *FoN* follicular neoplasm, Oncocytic cell-type, *SFM* suspicious for malignancy, *HTT* hyalinizing trabecular tumor, *GD* graves’ disease, *IPL* intrathyroid parathyroid lesion, *PM* positive for malignancy, *OFN* oncocytic follicular neoplasm, *PTC* papillary thyroid carcinoma, *FVPTC* follicular variant of PTC, *NIFTP* noninvasive follicular neoplasm with papillary-like nuclear features, *MTC* medullary thyroid carcinoma, *FTC* follicular thyroid carcinoma, *OTC* oncocytic thyroid carcinoma, *PDTC* poorly differentiated carcinoma, *ATC* anaplastic thyroid carcinoma, *OTC* oncocytic cell carcinoma. * including oncocytic variant of PTC

**Table 2:** Frequently encountered thyroid entities with tricky morphology

	Tricky morphology
ND	Scant cells Minimal reactive cells
Benign	none
HT	Limited oncocytic cells Reactive features misdiagnosed as PTC
Cystic lesions	Large and complex cysts Atypical repairing cyst-lining cells Squamous cells
Squamous cells in thyroid FNAC	Frequently associated with benign entities Frequently squamous metaplasia in thyroid entities Malignant squamous morphology in malignant entities
Graves’ Disease	Morphological atypia due to the treatment resembling malignant features Importance of clinical history
PTC	Nuclear pseudoinclusions can be seen in several different entities Not only peculiar of PTC
PTC subtypes	It is not necessary to diagnose them on cytology Warthin-like PTC subtype may mimic HT Tall-cell PTC subtype show spindle cells seen in other entities too

*ND* non-diagnostic, *HT* hashimoto thyroiditis, *PTC* papillary thyroid carcinoma

**Table 3:** Rare thyroid entities with tricky morphology

	Tricky morphology
IPL	Frequently misdiagnosed as follicular/oncocytic lesions Scant cytoplasm and nuclear molding are an important clue Posteriorly located is an important clue ICC necessary
HTT	Nuclear pseudoinclusions resembling PTC Stromal hyaline materiale resembling amyloid in MTC spindle-fusiform nuclei resembling MTC MIB 1 expression PAX8::GLIS 3 in 100% of cases
PDTC	Different architectural pattern including microfollicular resembling a follicular neoplasm **Mild atypia** **ICC to exclude other entities**
DHGTC	Difficult to identify mitotic figures and necrotic component
MTC	Different architectural patterns Variable cellular shapes Nuclear pseudoinclusions Mimicking many entities Crucial the support of IHC
ATC	High grade morphology Difficult diagnosis from a metastasis
Paraganglioma	Microfollicular pattern resembling a follicular neoplasm Nuclear pseudoinclusions resembling PTC stippled chromatine and granular cytoplasm resembling MTC
ITL	Very rare Lymphocytes and spindle cells Malignant morphology in carcinoma IHC highly recommended
Metastases	Variable morphology depending of the primary tumor ICC essential
Lymphoma	Difficult identification of atypical lymphocytes on the smears Need for Flow cytometry
Cribriform morular carcinoma	Resembling PTC Need for immunomarkers
sarcoma	Very rare Resembling spindle cell entities Need for immunomarkers

*IPL* intrathyroid parathyroid lesion, *HTT* hyalinizing trabecular tumor, *ICC* immunocytochemistry, *PDTC* poorly differentiated carcinoma, *DHGTC* differentiated high-grade thyroid carcinoma, *MTC* medullary thyroid carcinoma, *ATC* anaplastic thyroid carcinoma, *ITL* intrathyroid thymic lesions

Due to the worldwide adoption of TBSRTC, this review discusses the spectrum of thyroid lesions that can be diagnosed on FNAC classified according to TBSRTC categories. This review also covers important diagnostic pitfalls that can lead to false positive and false negative thyroid FNA results and assesses the role of ancillary techniques that might be helpful in reaching a conclusive diagnosis.

### Non-diagnostic thyroid FNAS

The definition of non-diagnostic (ND) specimens is based on thyroid samples that are inadequate for cytological interpretation in TBSRTC [8]. This diagnosis is the result of many different factors including the nature of the nodule (cystic vs solid vs fibrotic and calcified), as well as technical issues of the FNA procedure and operator experience. The best recommendation for limiting the rate of ND results, is a repetition of the FNA under sonographic guidance (US) in order to identify specific areas inside the nodule. Data from the literature showed that a repetition is associated with a significant reduction (from 70 to 83%) in the ND rate [25–28]. TBSRTC reports a risk of malignancy (ROM) of 13% for ND samples, ranging from 5 to 20% [8]. It is important to remember that a ND sample can be tricky especially because PTC has been reported as the most frequent cause of a false negative (FN) diagnosis in the ND category [25–28].

It is well-known that the presence of rare, isolated follicular cells with cytologic atypia, in the evaluation of a thyroid FNAC which might otherwise be interpreted as ND, should rather be reported as AUS, even without the required minimal adequacy number of follicular cells [8].

### Cystic lesions

Hence, other easy, but sometimes tricky sample to evaluate, using cytomorphology alone are the cystic or predominantly cystic thyroid nodules [15, 23, 25–28]. A cystic nodule can be seen in approximately 15–25% of thyroid lesions, usually corresponding to a histologically benign diagnosis [25–28]. Even though a typical benign cystic lesion is easily identified by the microscopic findings of foamy and hemosiderin-laden macrophages, scant colloid, and few follicular cells, it may still be associated with both FN and FP results [15, 23, 28]. In general, for cystic lesions smaller than 3 cm, the ROM is generally low. However, the ROM, especially for a possible cystic PTC, rises with larger and more complex cystic patterns [26–30]. Of note, atypical cells in benign cystic lesions can be derived from cyst-lining cells undergoing epithelial repair [15, 26–31]. Their identification is based on the following morphological findings: a) well-defined cellular borders, b) dense granular cytoplasm, c) enlarged nuclei with regular nuclear borders and d) occasional pale nuclei. A diagnosis of AUS-other, according to the 3rd edition of TBSRTC, is considered more appropriate for these cases [8].

Another diagnostic issue, leading to a possible pitfall is the detection of rare and occasional, or diffuse squamous cells, which can be encountered in thyroid FNAs [28–30]. Specifically, squamous cells can be encountered in many different entities including benign lymphoepithelial cysts, epidermoid cyst, thyroglossal duct remnant, and squamous metaplasia in long-standing HT. Infrequently, squamous cells may reflect a malignant lesion such as PTC, squamous subtype of ATC, metastatic squamous cell carcinoma, mucoepidermoid carcinoma (MEC), or carcinoma with a thymus-like differentiation [28–31]. Nonetheless, the presence of bland squamous cells, associated with a lymphocytic background and abundant anucleated keratin material more likely favors a benign cystic entity. It is important to remember that reactive squamous metaplasia is typically seen in HT [32]. Moreover, malignant squamous cells are usually easily recognized based on the morphological malignant features [29–32].

### Benign category

In many cases it is easily recognized that benign lesions are characterized by watery colloid associated with thyrocytes arranged as sheets or clusters, which are classified as benign according to TBSRTC and any other classification systems [4–8] (Table 1).

Furthermore, a FNAC sample with a diffuse population of oncocytic cells arranged in flat sheets or as isolated cells in a polymorphous lymphocytic background, as well as in a lympho-epithelial cluster, is diagnosed as a typical case of Hashimoto thyroiditis (HT). However, according to the different phases of HT, FNAC specimens can result in a range of diagnostic features leading to potential pitfalls [31, 32]. In fact, the prevalence of either the lymphoid or oncocytic component in HT may raise the possibility of a lymphoma or an oncocytic cell neoplasm, respectively. It is crucial to recognize that in HT, oncocytic cells can exhibit nuclear enlargement, grooves and chromatin clearing leading to a possible false positive (FP) interpretation as papillary thyroid carcinoma (PTC) [33–35]. As a rule, FNAC samples with a prominent population of lymphoid cells where lymphoma is a concern, should ideally be evaluated with the support of flow cytometry [33, 34]. Similarly, a prevalence of oncocytic cells might suggest different interpretations including oncocytic metaplasia in HT, oncocytic AUS or a true oncocytic follicular neoplasm [33–35].

Among the common benign entities that can be diagnosed with FNAC is Graves’ disease (GD), which has the same low ROM (between 1.9% and 2.5%) of other benign lesions. Nonetheless, some treated patients with GD might show some morphological features that could raise a suspicion for a PTC [24, 35, 36]. It is important to remember, in order to avoid pitfalls, that the classic cytologic features of a GD sample include aspirates composed of scattered follicular cell pleomorphism, fire-flare cells, oncocytic cell changes, and background lymphocytes. Furthermore, radioiodine treatment might induce nuclear and cellular enlargement, anisonucleosis, coarse chromatin, hyperchromasia and cytoplasmic vacuolization, with the lack of fine powdery chromatin. To avoid a FP diagnosis, it is crucial in such cases to have clinical information about the patient’s GD clinical history [36].

### Granulomatous thyroiditis

Granulomatous or De Quervein thyroiditis (GT) is defined as a self-limiting inflammatory associated with clinical evidence of neck and ear pain and tenderness occurring a few weeks after a viral upper respiratory tract infection [37, 38]. Due to the different stages of the disease, the cytological features are variable. In the initial phase, neutrophils and eosinophils are dominant, resembling an acute thyroiditis; the later stages show hypocellularity with giant cells, epithelioid cells, lymphocytes, macrophages, and scant degenerated follicular cells. In the involutional stage, one might find only giant cells and inflammatory cells. However, as suggested by Solano et al. the criteria for a GT include: presence of follicular cells with intravacuolar granules and/or plump transformed follicular cells; epithelioid granulomas; multinucleated giant cells; an acute and chronic inflammatory background; absence of fire-flare cells, hypertrophic follicular cells, oncocytic cells, and transformed lymphocytes. GT can mimic a variety of entities such as hemorrhage and infarction in a nodular goiter, and final stages of HT [37, 38].

### Indeterminate lesions-including atypia of undetermined significance (AUS) and follicular lesions (FN)

One of the most important challenges in the field of thyroid cytology is represented by the “gray zone” of indeterminate thyroid FNA lesions representing at around 20%-25% of all FNAC. A conclusive interpretation of indeterminate lesions is challenging leading to some unnecessary surgical resections (lobectomy or total thyroidectomy) [8, 21, 39, 40]

The atypia in AUS/FLUS includes a range of nuclear and/or architectural changes [8, 14, 21, 39, 40]. The 3rd edition of the Bethesda subclassified AUS as nuclear atypia and other, defining all the morphological criteria [8]. It stands to reasons, that both nuclear and architectural patterns can result in diagnostic pitfalls even though a majority of these findings are due to benign conditions such as hyperplastic-adenomatous nodules, toxic adenomas, and chronic lymphocytic thyroiditis. Two benign instances where AUS/FLUS diagnosis can be avoided include the presence of few oncocytic cells and/or cyst-lining cells with mild atypia mixed with benign follicular cells, and the presence of papillary structures without any nuclear features of PTC [8].

Furthermore, the 3rd edition of TBSRTC differentiated, based on cytomorphological criteria alone the diagnose either atypia of undetermined significance (AUS) with nuclear atypia or other lesions (AUS-other) from follicular neoplasm (FN) [11]. However, the exact etiology (benign or malignant) of these two categories of AUS lesions cannot be assessed on cytomorphology alone, which is why TBSRTC underlined, among the management options, that ancillary molecular testing should be often prescribed in the workup of these indeterminate thyroid nodules [8].

**Fig. 1:**
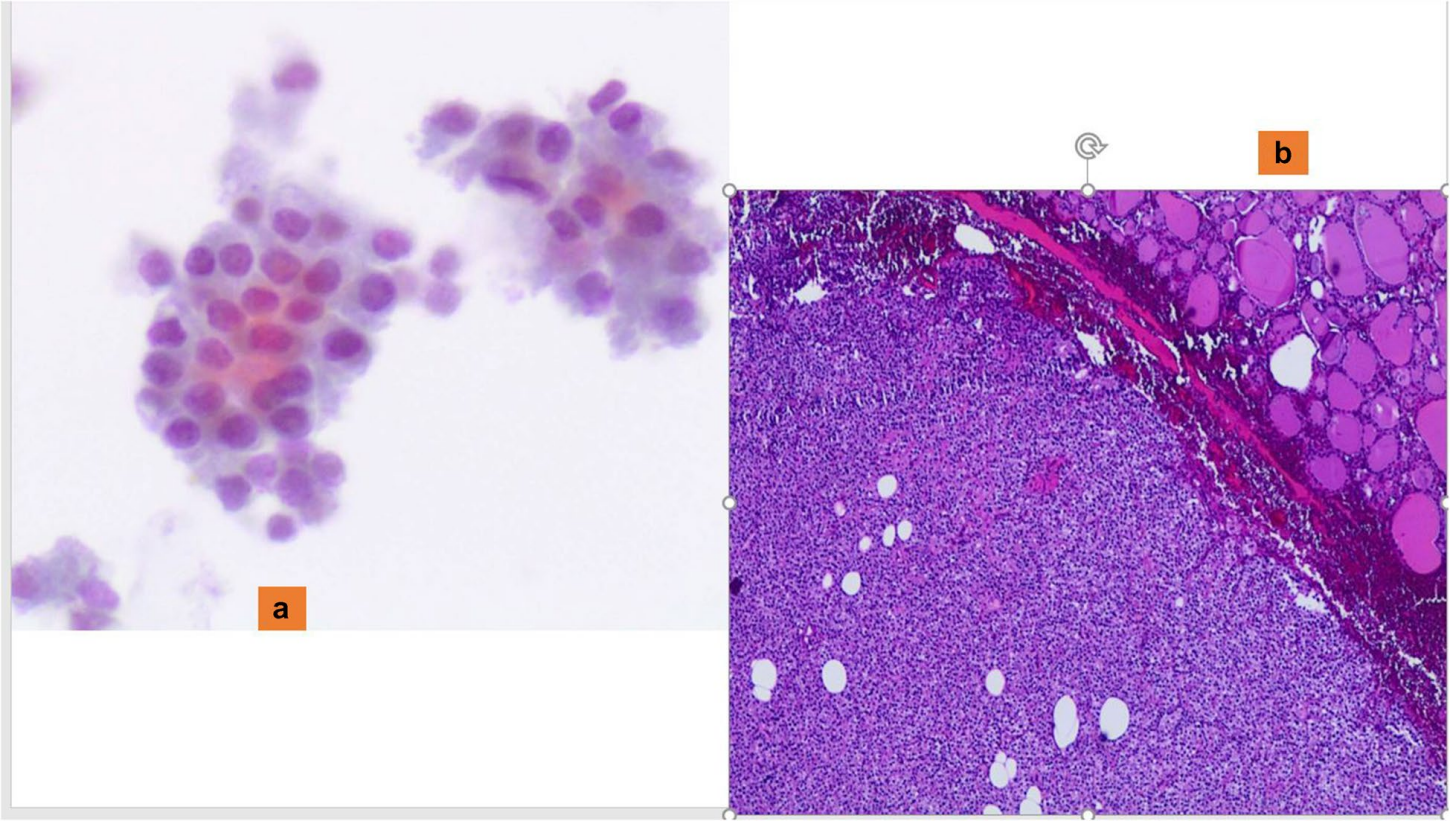
**a** The morphological detail of clusters of parathyroid intrathyroid cells, characterized by granular cytoplasm and round and dark nuclei (Pap stain 200x). **b** The details of an intrathyroid parathyroid adenoma with regular borders (H&E 200X)

Intrathyroidal parathyroid adenoma, composed of either chief (Fig. 1a and b) or oncocytic cells, is often misclassified as a follicular or oncocytic neoplasm, respectively [41, 42]. Many of these FNAC samples show a predominantly microfollicular pattern, which is very similar to the epithelial clusters of a follicular neoplasm. Nevertheless, the combination of few details including the evidence of a posterior nodule detected on ultrasound with the morphological features of epithelial cells with small hyperchromatic nuclei, scant cytoplasm, forming crowded trabecular clusters, and with frequent nuclear molding without colloid are suspicious for the diagnosis of a parathyroid neoplasm [41, 42]. However, it is essential to confirm the parathyroid origin of such a lesion, the support of either an immunostain positive for parathyroid hormone (PTH) and/or molecular analysis showing the presence of the PTH gene can be helpful. [8, 41, 42]. In these cases, thyroglobulin and TTF1 are negative.

**Fig. 2:**
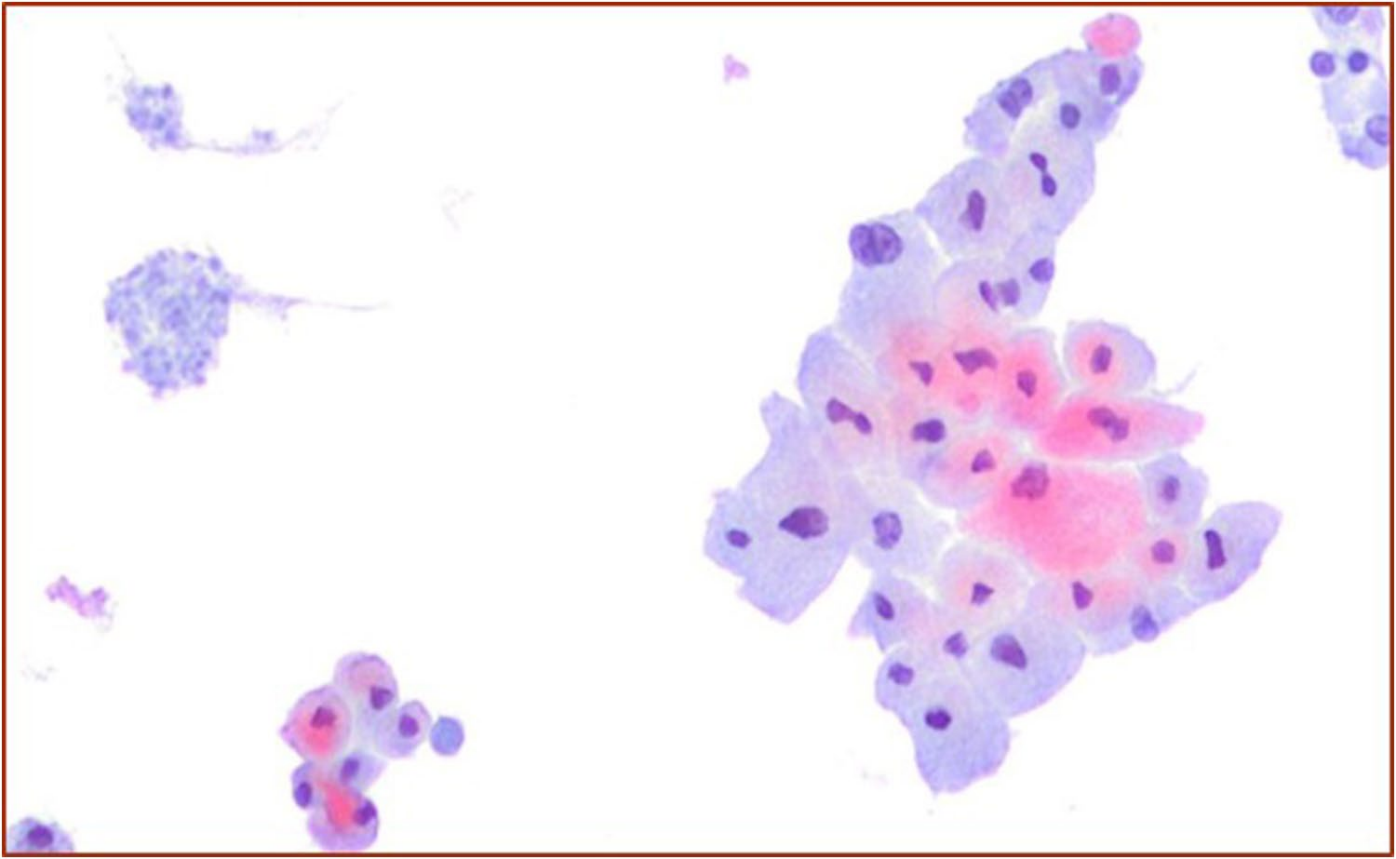
The details of oncocytic cells in an oncocytic neoplasm (Pap stain 200x)

This diagnostic category also includes some entities such as NIFTP, follicular adenoma versus follicular carcinoma, and oncocytic adenoma versus oncocytic carcinoma [8] which cannot be further classified on FNA (Fig. 2). This is due to the evidence that these entities show significant morphological overlapping findings between benign versus malignant counterparts, combined with the impossibility to identify any type of intra-parenchymal infiltration including capsular or vascular invasion [8, 43–47].

**Fig. 3:**
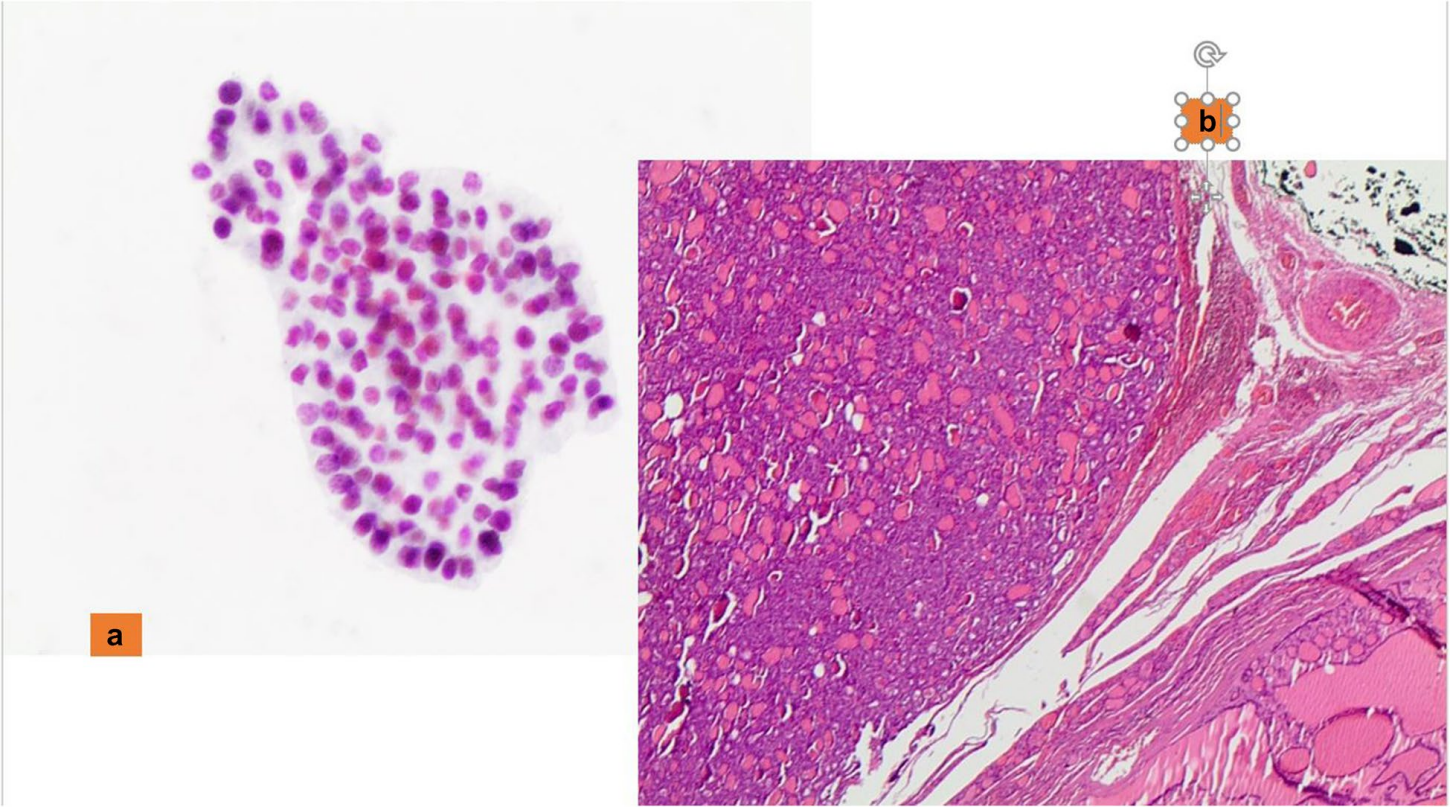
**a** A cluster of follicular cells with irregular nuclei and atypical features diagnosed as suspicious for malignant. **b** The histological criteria of a follicular-patterned lesion with the nuclear features of PTC, lack of capsular and vascular invasion, diagnosed as NIFTP (H&E 200X)

The distinction between NIFTP and the other follicular patterned neoplasms is impossible to make solely on cytology, and even with the support of ancillary techniques. Since the introduction of NIFTP, several studies have provided insight on the impact of this newer terminology on the interpretation of thyroid lesions, showing that the majority of NIFTP cases are often classified in TBSRTC categories III, IV and V [8, 43–47] (Fig. 3a and b). According to TBSRTC, when the features of a follicular neoplasm are encountered with mild/bland atypia or nuclear clearing, a comment including NIFTP among the differential diagnoses is the best choice. Most importantly, is to avoid the diagnostic pitfall of interpreting NIFTP as PTC, which may arise in those cases that are follicular patterned but lack papillary structures.

The distinction between follicular adenoma and follicular carcinoma, including those tumors composed of only/almost exclusively of oncocytic cells (Fig. 2) is impossible on thyroid FNAC [8, 33–36]. As for the discrimination between NIFTP versus encapsulated minimally invasive follicular variant of PTC, a thyroid cytology sample alone is not able to define any capsular and vascular invasion. Recently, some investigators have highlighted a higher association between positivity for a dedicated immunopanel (including HBME-1, Galectin-3 and CK-19 among the others), and the malignant nature of follicular-derived neoplasms [33–39]. However, the detection of mitotic figures, a necrotic component, as well as atypical and pleomorphic nuclei might help in reaching the correct diagnosis [8].

### Oncocytic follicular neoplasms

The term onocyctic cells is used to define thyroid follicular cells with abundant granular mitochondria-rich cytoplasm and they have been found in several conditions [36, 37] including adenomatous/hyperplastic nodules, chronic lymphocytic (Hashimoto) thyroiditis, multinodular goiter, but as well as oncocytic adenoma and carcinoma [31–34]. A common diagnostic pitfall is misinterpreting the presence of a population of oncocytic cells in cluster admixed with background colloid with or without sheets of non-oncocytic follicular cells [36, 37] as Oncocytic neoplasm, whilst the presence of both component is in favor of a benign nodule.

In difficult and controversial cases of lymphocytic thyroiditis, especially when the lymphocytic component is scant, a clue to the correct interpretation is based on finding oncocytic cells organized in small clusters of 3–10 cells with large nuclei, with or without glassy chromatin and with nuclear features that may raise concern for PTC. A high threshold should be maintained when background lymphocytes are present [36, 37]. Another difficult differential diagnosis is between Oncocytic neoplasms and the oncocytic variant of PTC, is especially challenging and mostly based on the identification of the PTC nuclear criteria. It is also to underline that some oncocytic neoplasms are likely to resemble MTC which is frequently composed of dispersed cells with eccentric nuclei and abundant dense granular cytoplasm [48–50]. A subtle clue is that MTC nuclei do not show the widespread presence of prominent nucleoli characteristic of oncocytic cells. The conclusive diagnosis is supported by the application of an IHC panel, including thyroglobulin, calcitonin, CEA, and chromogranin can be useful.

It is important to highlight that also an intrathyroidal oncocytic parathyroid nodule (including both adenomas and carcinomas) is an important cytological pitfall [41, 42]. Nonetheless, oncocytic parathyroid samples are characterized by a monomorphic population of cells, with small round nuclei, and more condensed chromatin pattern than in medullary carcinoma. A specific immunoprofile showing positivity for parathormone (PTH), while being negative for thyroglobulin, calcitonin, and TTF-1 supports the diagnosis of a parathyroid neoplasm.

### Papillary thyroid carcinoma (PTC)

Among the malignancies that are easily recognized by cytomorphology alone in FNAC, there are samples with cells showing nuclear and cellular criteria characteristic of PTC [8, 51, 52]. In fact, if the classical papillary architecture, large clusters of pleomorphic cells with typical PTC nuclear features (grooves, nuclear pseudoinclusions, pale nuclei, powdery chromatin), thick bubble-gum colloid, and psammomatous bodies are present, they easily lead to a diagnosis of PTC [8]. However, when there are limited and subtle cytologic findings, they can lead to diagnostic pitfalls, including both FP and FN results [8]. Specifically, it is important to underline that while the detection of intranuclear cytoplasmic pseudoinclusions (INCIs) is seen in the majority of PTC cases, they can also arise in other benign and malignant entities including medullary thyroid carcinoma (MTC), poorly differentiated thyroid carcinoma (PDTC), ATC, hyalinizing trabecular tumor (HTT), noninvasive follicular thyroid neoplasm with papillary-like nuclear features (NIFTP), and rarely other benign nodules [51–53].

The different subtypes of PTC might cause diagnostic pitfalls [51–53]. Of note, the different classification systems, including TBSRTC, do not recommend diagnosing the subtypes of PTC based solely on cytology samples [8, 51–53].

**Fig. 4:**
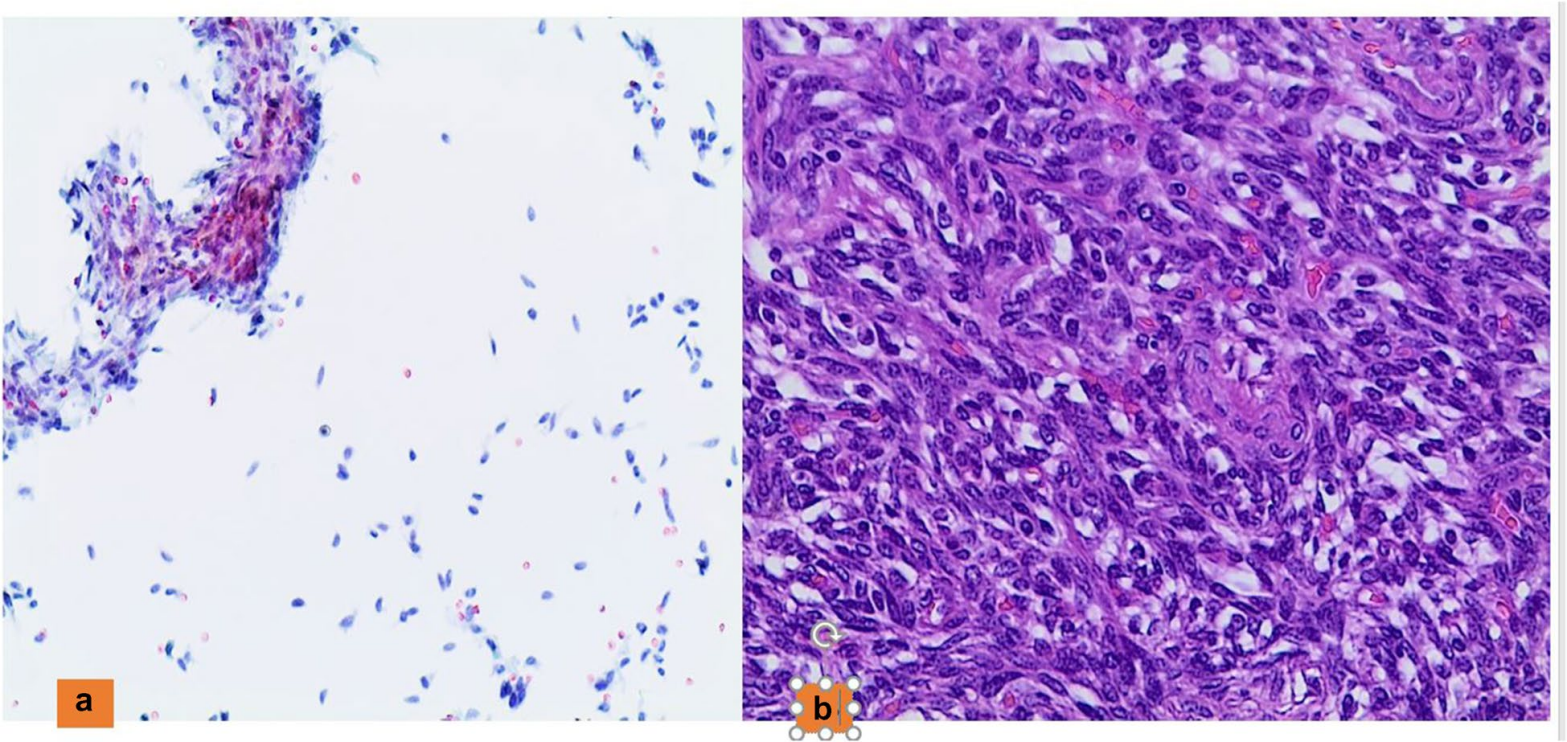
**a** The cytological details of a solitary fibrous tumor (PAP stain 200x). **b** The histological details of the same case (H&E 200X)

However, among the subtypes of PTC, the Warthin-like subtype may mimic HT, even though such samples have more pleomorphic and irregular nuclear membranes, nuclear pseudoinclusions, and exhibit less prominent nucleoli than oncocytes seen in HT. In addition, the Warthin-like variant of PTC shows permeating lymphocytes within epithelial groups and background plasma cells. Furthermore, the “tall cell” subtype of PTC might show some spindle and/or elongated nuclei which can be seen in many other entities such as MTC, HTT, Solitary fibrous tumor-SFT (Fig. 4), Spindle Epithelial Tumor With Thymus-Like Differentiation (SETTLE) and sarcoma [54–56].

Hyalinizing trabecular tumor (HTT) is a common cause of a FP diagnosis of PTC [8, 56–60]. The reason for its misinterpretation is attributed to the rarity of this tumor and its associated morphological features of trabecular growth, marked stromal hyalinization, and nuclear grooves with pseudoinclusions leading to a misdiagnosis of either PTC and/or also MTC [8, 56–60]. However, a careful evaluation of the cytological samples shows that HHT, compared with PTC, demonstrates the lack of papillary architecture as well as powdery and pale nuclei with elongated epithelial cells which are typically encountered in PTC, together with acellular stromal hyaline material (Table 4). The support of ancillary studies, including cytoplasmic immunopositivity for MIB-1, the lack of *BRAF*^*V600E*^ mutation and the presence of specific *PAX8::GLIS3 or GLIS1* (virtually 100% of the cases) rearrangements are able to resolve the issue and make the diagnosis [60].

**Table 4:** Thyroid diagnoses supported by immunocytochemistry

	Positive staining	Negative staining
IPL	PTH, Galectin-3, GATA-3	TTF1, Thyroglobulin, PAX8
OFN	Anti-mitochondria TTF1 Thyroglobulin PAX8	
PTC	HBME-1, CK-19 Galectin-3 TTF1 Thyroglobulin PAX8	
MTC	Calcitonin, CEAm, INSM1	Thyroglobulin
PDTC	TTF1, Thyroglobulin, PAX8, P53	
ATC	CAM 5.2	TTF1, Thyroglobulin
METASTASES	Based on primary tumor	Based on primary tumor

*IPL* intrathyroid parathyroid lesion, *OFN* oncoytic follicular neoplasm, *PTC* papillary thyroid carcinoma, *MTC* medullary thyroid carcinoma, *PDTC* poorly differentiated carcinoma, *ATC* anaplastic thyroid carcinoma

### Poorly differentiated thyroid carcinoma (PDTC)

**Fig. 5:**
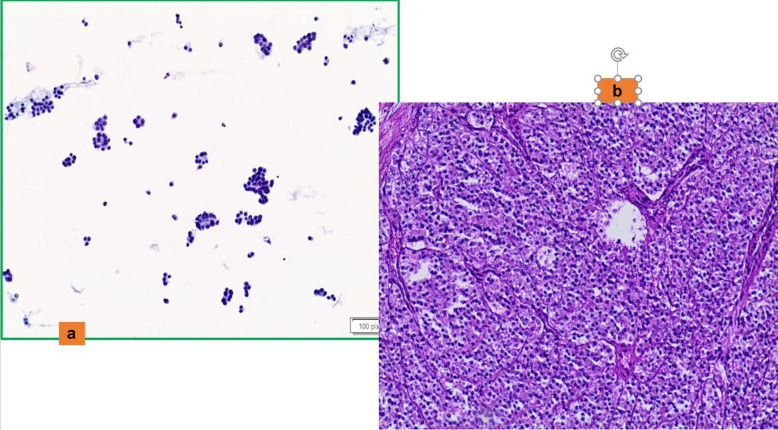
**a** The morphological detail of a microfollicular proliferation of cells which were diagnosed as Follicular neoplasm (Pap stain 200x). **b** The morphological details of a lesion showing a poorly differentiated component on histology (H&E 200X)

**Fig. 6:**
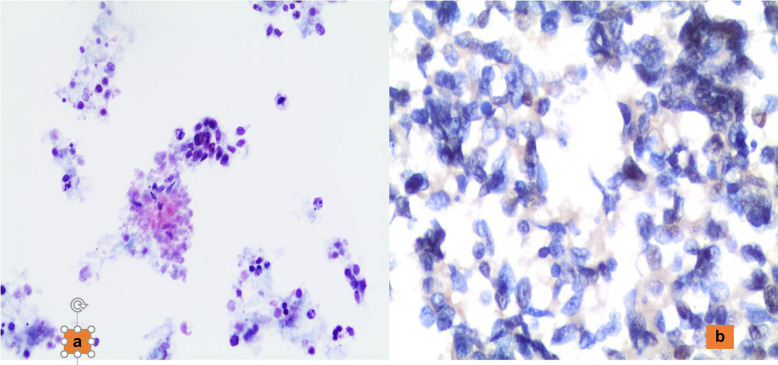
**a** The cytological component of a small cell lung carcinoma metastasizing on the thyroid. **b** The expression of thyroglobuline in those cells (Pap stain 200x; AB200X)

Another tricky diagnosis is represented by poorly differentiated thyroid carcinoma (PDTC). PDTC is defined as a thyroid carcinoma composed of follicular cells organized in an insular, solid, or trabecular growth pattern [8, 61, 62] Cytologically, PDTCs are difficult to recognize mostly due to the fact that their cytomorphological features are characterized by mild atypia and clusters of cells with a microfollicular pattern (Fig. 5a, b), thereby resulting in these tumors frequently being misclassified as a follicular neoplasm [62, 63]. However, the presence of apoptosis, mitotic activity, anisonucleosis, and necrosis combined with an insular and/or trabecular pattern of monomorphic atypical follicular cells that have an increased nuclear/cytoplasmic ratio might suggest a diagnosis of suspicious for malignancy, non-otherwise specified [8, 61, 62]. Furthermore, PDTC might show a predominance of isolated single cells with a discohesive architectural pattern leading to an erroneous diagnosis of MTC, possible metastatic tumor (Fig. 6), or lymphoproliferative disorder. The latter diagnoses can be excluded with the support of ancillary techniques [8, 61, 62].

### High-grade differentiated thyroid carcinoma (DHGTC)

**Fig. 7:**
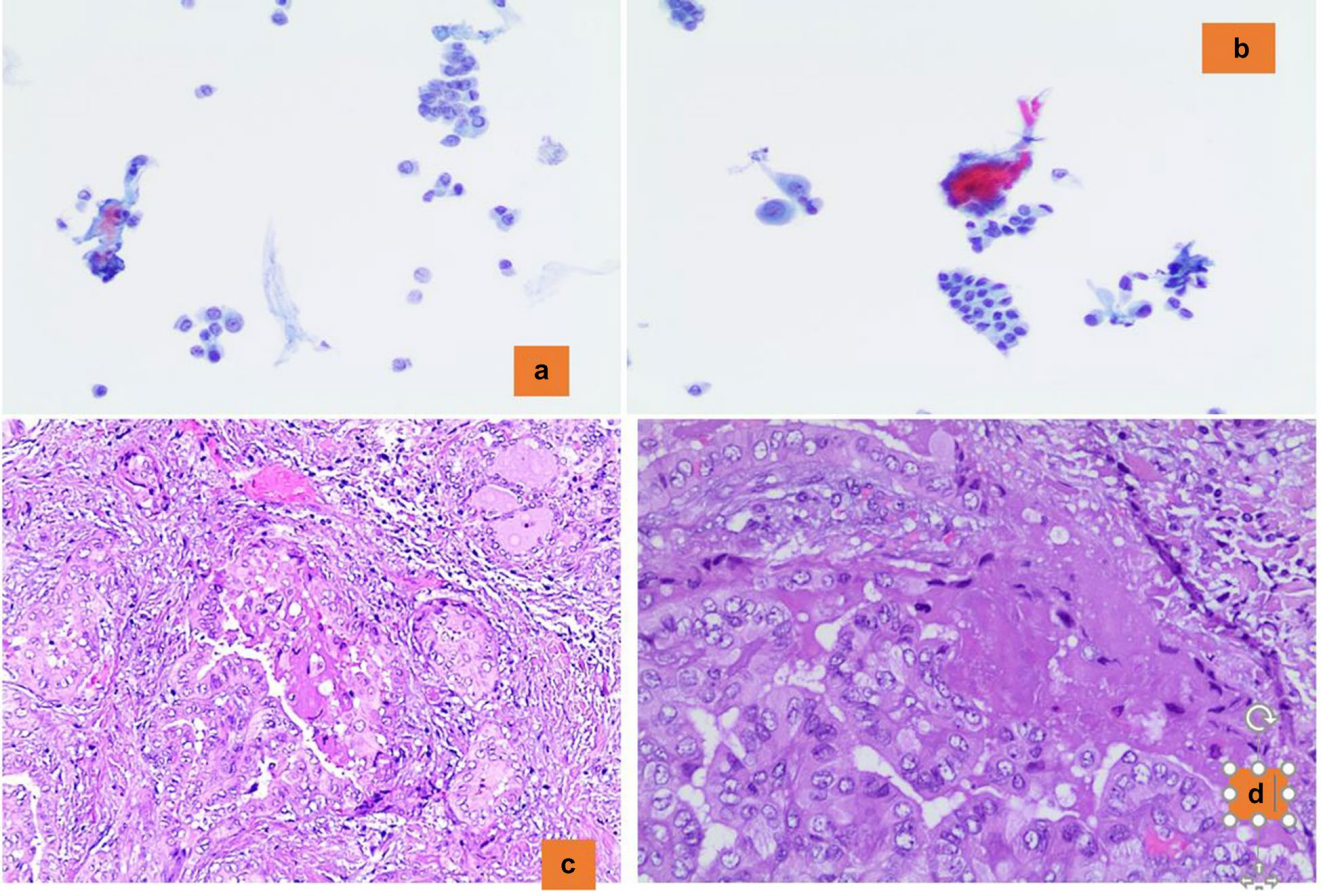
**a**, **b** The morphological detail of a malignant lesion, mostly showing the features of papillary carcinoma, with focal necrotic component (Pap stain 200x). **c**, **d** The histological details of a differentiated high-grade carcinoma with necrotic component (H&E 200X)

The WHO classification scheme of thyroid neoplasms, 5th edition (2022) introduced a new entity termed high-grade differentiated thyroid carcinoma (DHGTC) [63, 64]. DHGTCs are still differentiated cancers based on the fact that they retain the distinctive architectural and/or cytologic findings of well-differentiated histotypes of carcinoma of follicular-cell derivation showing at least one of these additional findings: necrotic component within the tumor and/or ≥ 5 mitotic figures per 2mm^2.^ The recognition of this new entity has an implication for its cytological diagnosis. Although the identification of DHGTC as a malignant lesion is easy to acknowledge, interpretation as a “DHGTC” requires evidence of either a necrotic component and/or mitotic figures. A recent multi-institutional study including 40 cases with prior fnac. the results showed that the majority of them were PTC (65%), followed by FTC (22.5%), and OTC (12.5%) [63]. On cytology, over 97% of FNA cases were classified as Bethesda category IV or above. In 25% of them, there were marked malignant features including marked cytologic atypia, increased anisonucleosis, large oval nuclei, mitotic activity, or necrosis (p < 0.05). Furthemore, 68% of DHGTC cases were associated with high-risk molecular alterations. *TERT* promoter mutations occurred in 41%, of which 89% of these were associated with a second mutation, usually RAS or BRAF p.V600E. The detection of necrotic component, mitotic figures and marked pleomorphic findings are not always identifiable in cytological samples, making the diagnosis of the DHGTC entity very challenging (Fig. 7a–d).

### Medullary thyroid carcinoma (MTC)

**Fig. 8:**
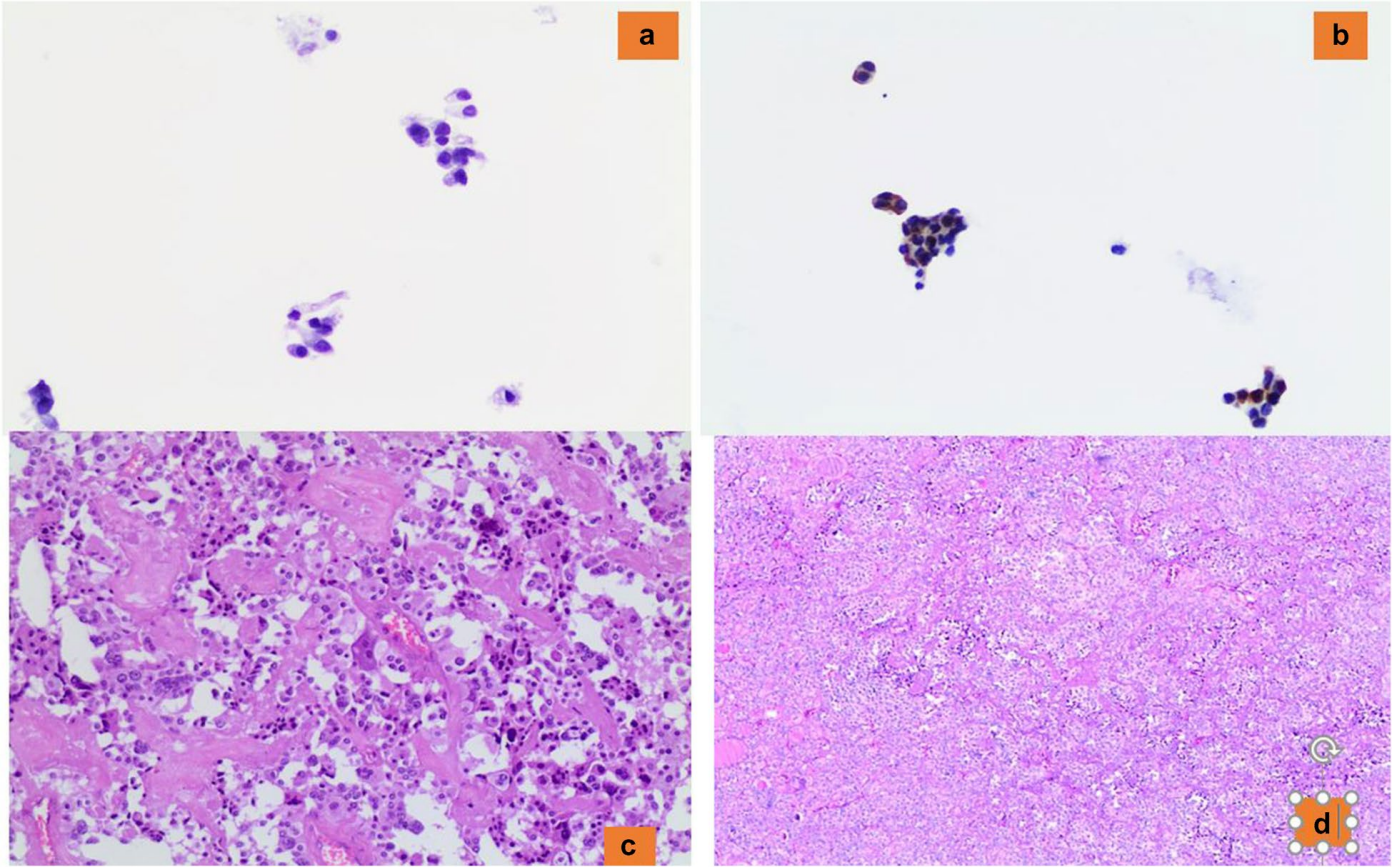
**a** The morphological detail of a medullary thyroid carcinoma (Pap stain 200x). **b** The positivity for calcitonin (AB 200X). **c**, **d** The histological details of a medullary thyroid carcinoma (H&E 200X)

The detection of a cellular cytology sample with isolated cells and/or a syncytium-like pattern of cells with variable shape (spindle, epithelioid, plasmacytoid, clear cells) and eosinophilic cytoplasm is highly suggestive of MTC [8, 47–50] (Fig. 8a–d). Nonetheless, for a definitive diagnosis of MTC, it is crucial to obtain support utilizing immunostains such as positivity for Calcitonin, CEAm, INSM1, and negativity for thyroglobulin [47–50]. It is well-known that MTC, mostly because of its numerous growth patterns, represents one of the trickiest mimickers to diagnose among thyroid entities. Not only is MTC sometimes difficult to recognize on histology, but it could be extremely challenging to interpret on misleading cytological samples [47–50]. According to the various growth patterns, the most important differential cytological diagnoses include oncocytic follicular neoplasm (with red cytoplasmic granules in MTC versus blue-gray cytoplasmic granules in oncocytic follicular neoplasm-OFN), PTC (with nuclear pseudoinclusions), PDTC, ATC and metastases, among others (Fig. 6a, b). To solve the issue, adequate aspirated material is required to perform an immunopanel of stains [49, 50].

### Anaplastic thyroid carcinoma

**Fig. 9:**
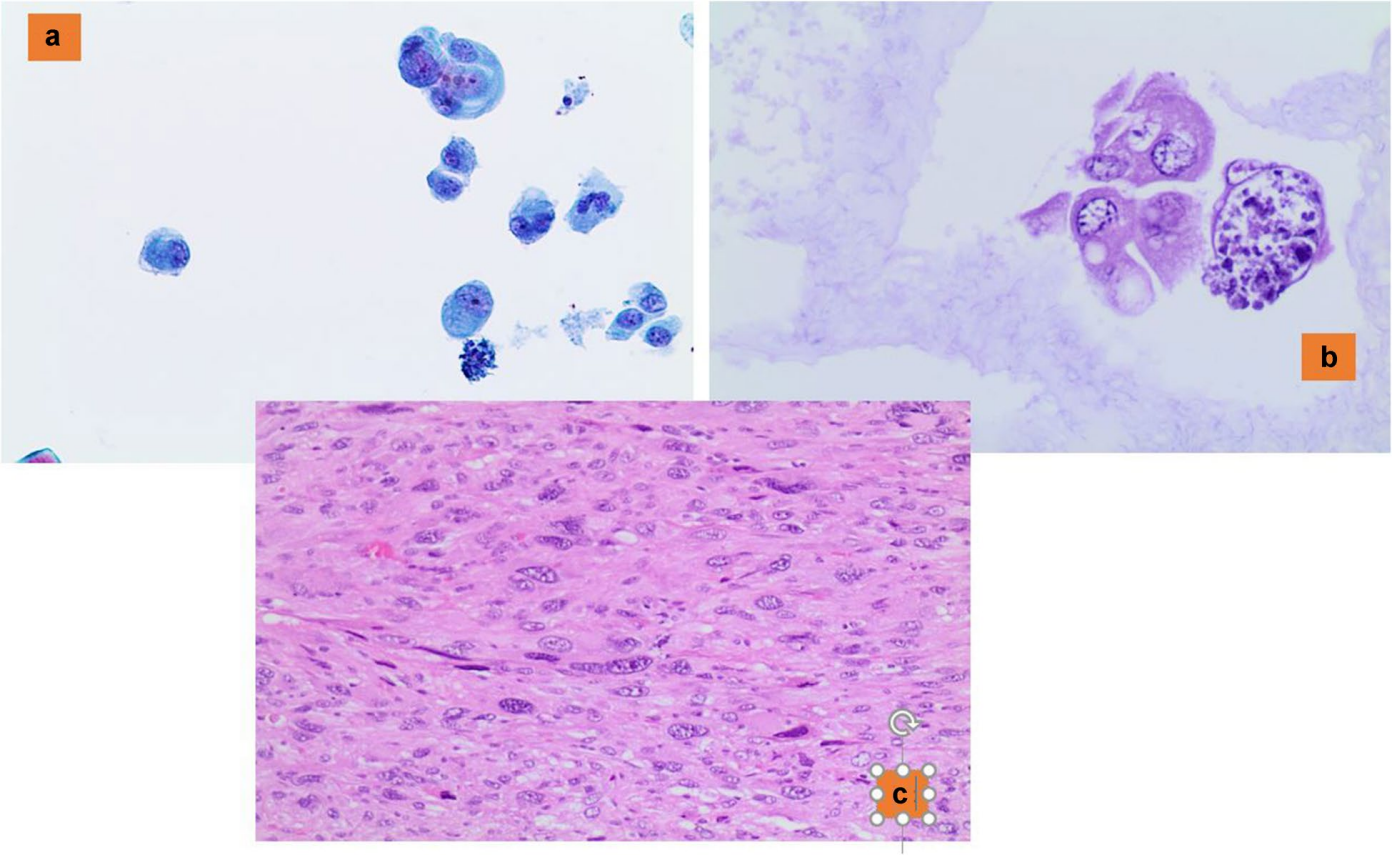
**a** The morphological detail of an anaplastic thyroid carcinoma with also necrotic component (Pap stain 200x). **b** The spindle pattern of an ATC on cell block (H&E 200X). **c** The histological details of an anaplastic thyroid carcinoma (H&E 200X)

ATC is an aggressive thyroid carcinoma, easily recognized in cytological samples as a high-grade malignancy composed of pleomorphic epithelioid and spindle shaped cells [8, 65] (Fig. 9a–c). Suspicion for ATC in an FNA sample is mostly based on a combination of cytomorphological findings in the clinical context of a rapidly growing mass in a hard, nodular thyroid gland, with infiltration identified on radiology imaging into surrounding extrathyroidal soft tissue. The morphological identification of high-grade undifferentiated cells in an FNA aspirate raises the differential diagnosis of metastatic malignant tumors, sarcoma, PDTC, MTC and lymphoma. The most common diagnostic pitfall observed when this scenario is encountered is a metastatic tumor such as melanoma, sarcomatoid renal carcinoma, squamous cell carcinoma, and poorly differentiated adenocarcinoma [8, 65]. In this situation the support of ancillary studies is warranted, especially IHC studies including PAX-8 and keratins [8, 65] (Tables 2 and 3). Most ATC samples are negative for TTF-1 and thyroglobulin, and a subset are even negative for keratins. The possibility of a metastasis to the thyroid gland should always be considered for those patients with a previous history of a malignant extrathyroidal neoplasm [8].

### Rare entities

**Fig. 10:**
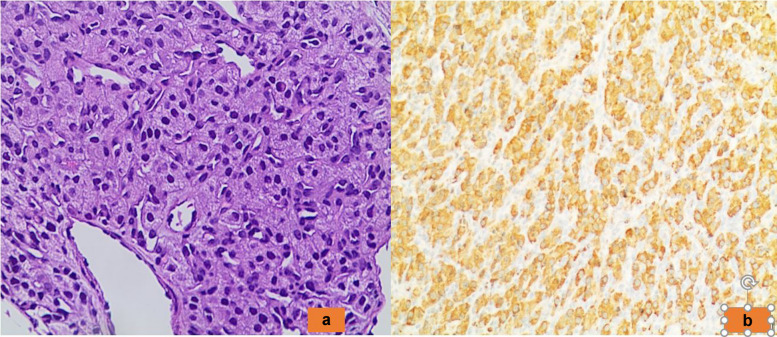
**a** The features of an intrathyroid paraganglioma (H&E 200x). **b** The expression of Cromogranin (AB 200x)

Intrathyroid paraganglioma represents another frequent pitfall, as it can be misdiagnosed as oncocytic neoplasm, PTC and MTC [56, 66]. The morphological features of this lesion mostly show specimens with cells arranged in clusters and a microfollicular pattern, vacuolated cells with poorly defined cytoplasm, nuclei with stippled chromatin, and occasionally nuclear pseudoinclusions (Fig. 10a, b). Another frequent misdiagnosis is with MTC sharing the stipple chromatine, and the eosinophilic granular cytoplasm. Nevertheless, paraganglioma shows immunopositivity for synaptophysin and chromogranin, together with negativity for thyroglobulin, Calcitonin, CEAM, which are helpful for reaching the correct diagnosis in doubtful cases.

The possibility to misdiagnose an intrathyroidal thymic lesion sampled by FNA is due to their rarity and overlapping cytomorphological features, mostly showing lymphocytes and spindle to fusiform cells [54, 55]. Intrathyroidal thymic carcinoma is mostly characterized by a dirty background combined with small clusters of poorly differentiated malignant cells with hyperchromatic nuclei [99–104]. For these thymic lesions, the support of immunostains is highly recommended, showing negativity for TTF1, thyroglobulin and calcitonin, whilst demonstrating immunopositivity for CD5 and keratins [54, 55, 67].

As already alluded to, another difficult lesion to sometimes accurately identify based solely on an FNA sample, is metastatic disease to the thyroid gland, especially when the patient does not have a history of a prior non-thyroid malignancy [8, 66, 68]. According to the literature, metastatic disease involving the thyroid gland can arise from a wide variety of different primary malignant entities. The cytomorphological pattern identified in the thyroid metastasis frequently resembles that found in the distant primary tumor. However, the correct diagnosis is typically based on a combination of cytomorphology, clinical history and application of ancillary techniques [8, 66, 68]. When the morphological features do not match with the classic primary thyroid entities. It is useful to have adequate material in order to perform a large panel of immunostains.

Another rare entity is represented by cribriform morular carcinoma. Since the last edition of the endocrine WHO 2022, this entity was included among the PTC subtypes [8, 67]. However, Cribriform-morular thyroid carcinoma (CMTC) was traditionally considered as a variant of papillary thyroid carcinoma (PTC); recent studies, however, showed that CMTC constitutes a clinicopathologically distinct category of thyroid carcinoma driven by Wnt / beta catenin pathway activation. The cytological features include: cellular smears with lack of colloide, tall, columnar neoplastic cells with a papillary-like arrangement and/or empty spaces formed by spindle to ovoid cells within cell clusters are present (cribriform pattern), with also the formation of morules. Nuclear grooves are present but intranuclear pseudoinclusions are less common than in the conventional papillary thyroid carcinoma. Cell block, if available, is extremely valuable to perform confirmatory immunostains including beta catenin. Among the differential diagnoses, PTC subtypes, PDTC, intestinal and breast carcinomas should be considered.

Among the very rare entities, a mention should be done for primary angiosarcoma of the thyroid, seen mostly in elderly in Alpine regions of Europe, where tumor may comprise 16% of thyroid malignancies, due to high prevalence of iodine deficient goiter [8, 56]. It is a high grade malignant population of cells, usually poorly differentiated, with irregular slit vascular spaces with anastomosing channels or discrete cytoplasmic vacuoles. The epithelioid variant has poorly circumscribed growth in sheets / cords, intracytoplasmic lumina filled with red blood cells and it is composed of polygonal epithelioid cells with abundant eosinophilic cytoplasm, vesicular nuclei, prominent amphophilic-basophilic nucleoli. The most important differential diagnosis is represented by ATC or a secondary angiosarcoma to the thyroid. It is crucial the support of immunostains showing positivity for CD31, CD34, Factor VIII, Vimentin.

**Fig. 11:**
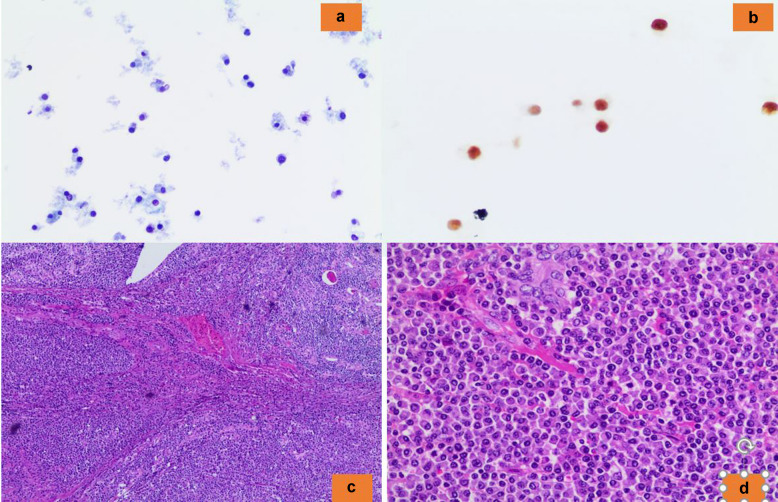
**a**, **b** The cytological details of a plasmocytoma in a and an expression of MUM1 in Fig. 11b (Pap stain 200x, AB 200X). **c**, **d** The histological details (H&E 200X)

A further rare entity is a primary plasmocytoma of the thyroid gland, which might mimic an OFN or/and an MTC [56]. The support of an immunopanel showing negativity for Keratin, Calcitonin, CEAm, and positivity for MUM1 and plasmacell markers is essential for the diagnosis (Fig. 11).

Concerning the spindle cell lesions, apart from PTC, MRTC and ATC that might show spindle-fusiform features we need to mention the rarer possibility of a solitary fibrous tumor or a mesenchimal lesion such as leyomioma or simply the fibroblatic riparative features after an FNA [8, 54–56]. The support of immunostain is crucial for the diagnosis, as well as we have a positivity for CD34, CD99, STAT6 for the SFT whilst positivity for Desmin and Actin for leyomioma. In both entities TTF1 and Thyroglobuline are negative, whilst they are positive in a follicular lesion with spindle features due to a prior FNA [8, 54–56]

### Pediatric thyroid lesions

Thyroid nodules are significantly different from the adult counterparts. The incidence of thyroid lesions in pediatric population is lower than adults ranging from 0.05 to 1.8%, even tough recent data showed that the incidence increased ranging from 0.6 to 2.7% [8, 69, 70]. Nonetheless, according to the Surveillance, Epidemiology, and End Results (SEER) database, the percentage of thyroid malignancy is increased 3% annually (lim). Specifically, pediatric thyroid cancers show several similarities to adult cancers, but also important differences in terms of molecular pathophysiology, clinical presentation, management and prognosis [8, 69, 70].

Furthermore, thyroid nodules are less commonly found in pediatric patients but with a higher ROM and frequently associated to prior radiation exposure, inherited syndromes, such as APC, DICER1 or PTEN mutations [8, 69, 70]. To note, the most common malignant entity is represented by PTC and its subtypes (90%), with some of them such as diffuse sclerosing type, typically encountered almost exclusively in childhood. The remaining are follicular cracinoma (FTC) representing at around 8–9%. Then the medullary thyroid carcinoma, originating from the parafollicular cells, accounting at around 4% [8, 69, 70].

It is important to underline that a child with suspicion of thyroid cancer should be referred to an experienced multidisciplinary thyroid team, mostly specifically dedicated to pediatric thyroid cancer. In this regard, TBSRTC 2023 introduced a specific diagnostic classification system for pediatric thyroid lesions with specific ROM and management. The pediatric classification maintained the same diagnostic categorization reported in adults with higher risk for each diagnostic category. Furthermore, as for the adult population, most issues are encountered in the diagnosis and management of the “grey zone” of indeterminate lesions, which however, show a higher number of malignancies at histology comparing with the indeterminate lesions in adults. The identification of the morphological criteria, combined with the support of ancillary techniques, is crucial for making a diagnosis and define the proper tailored management [8, 69, 70].

### Ancillary techniques

As reported per each diagnostic entity, the support of ancillary techniques, including immunocytochemical markers and molecular testings represent a valid additional diagnostic tool [71, 72] (Tables 4 and 5). In this perspective, the need for adequate material is crucial and significantly important. Some immunomarkers, are able to make a diagnosis of organs, discriminating between prior or metastatic localization to the thyroid, other markers are linked with a higher association with maliganncy, such as Galectin-3 and HBME-1 in PTC and its subtypes [71, 72]. The performance of these techniques can be carried out of liquid based cytology (LBC) stored material or on cell-blocks with feasible and reliable results in the majority of cases.

**Table 5:** More relevant molecular alterations in the thyroid entities

	Positive staining
OFN	PAX8::PPARG
FA/FTC	N, H, K-RAS; PAX8::PPARG
NIFTP	N, H, K-RAS; PAX8::PPARG
PTC	BRAFV600E, TERTp;
MTC	RET (mutations); H, N, K-RAS; rare gene fusions
High grade carcinoma of follicular pattern (PDTC and DHGTC)	TP53, TERT, PI3K, PTEN, AKT
ATC	TP53, TERT, PI3K, PTEN, AKT
METASTASES	Based on primary tumor

*OFN* oncoytic follicular neoplasm, *FA* follicular adenoma, *FTC* follicular carcinoma, *NIFTP* *NIFTP* noninvasive follicular neoplasm with papillary-like nuclear features, *PTC* papillary thyroid carcinoma, *MTC* medullary thyroid carcinoma, *PDTC* poorly differentiated carcinoma, *DHGTC* differentiated high grade thyroid carcinoma, *ATC* anaplastic thyroid carcinoma

Indeterminate cytology could benefit from further application of molecular testing. These test can be diagnostic and prognostic for thyroid nodules. Diagnostic thyroid molecular tests are designed primarily to further clarify preoperative determination of benign or malignant disease in patients with nodules with indeterminate cytology—most commonly, AUS or FN [8, 71, 72]. Although numerous diagnostic tests have been developed and piloted, few have undergone high-quality, multicentre, prospective validations with blinded histological review by different series. Among the others we include mutation, largely based on DNA data, RNA-based gene expression classifier, mostly used in USA and a molecular test available in the USA uses both microRNA expression and DNA mutation profiling [8, 71, 72].

Overall, molecular diagnostic testing should be considered in patients with clinically relevant thyroid nodules for which surgery is typically recommended on the basis of an indeterminate FNA cytology. These tests help avoid unnecessary surgery when other reasons for resection (eg, mass effect or cosmetic reasons) are not present. The choice of test in different countries depends on availability, cost, and other additional factors [8, 71, 72]. Considering PTC and its subtypes, the recurrent p. V600E missense mutation of the BRAF proto-oncogene is the most common genetic, and also, together with telomerase reverse transcriptase (*TERT*) promoter mutations, associated to a more aggressive clinical phenotype [71, 72]. The new frontier, in the field of immunocytochemistry, is the evidence that immunohistochemistry can be utilized instead as a surrogate screening tool for specific genetic events in PTC, including the mutation-specific BRAF antibody (clone VE1) to screen for V600E alterations, the pan-RAS Q61R (clone SP174) antibody that detects the most common HRAS/NRAS/KRAS Q61R mutations, as well as pan-TRK staining for NTRK1/3 fusions and the 5A4 and D5F3 antibodies optimized for the detection of ALK fusions [8, 71, 72]. Notably, all these immunomarkers have demonstrated excellent concordance with molecular screening results. It is important to underline that BRAF antibody identifies only the V600E alterations, but it does not stain lesions with other BRAF alterations, and its negativity does not exclude a thyroid wild type BRAF V600E carcinoma.

### Technical aspects

One of the major technical issues is defined by the artifacts due to previous FNAC whihc might lead to misinterpretation of the lesion in terms of fibrous tissue, hemorragic componente or fibroblastic proliferation of spindle cells. It is always necessary to combine the morphological features with the clinical history and the ultrasound pattern.

Also the performance of core needle biopsy might have some limitations mostly define by the possible interpretation of the nature of the tissue but offering limited information about the capsular and vascular infiltration in the field of indeterminate lesions. Its role seems to be more adequate in the evaluation of high grade malignancies such as ATC and the performance of an immunohistochemical panel. In this perspective a relevant role of FNA is defined by the possible setting of neoadjuvant therapies and prognostic significance.

Recently, ana emerging interest involves the idea of exploring the ability of a convolutional neural network (CNN) model to sub-classifying cytological images of Bethesda category IV diagnosis into follicular adenoma and follicular carcinoma [73]. Few papers invetsigated this aspect concluding that CNN model has achieved high sensitivity in recognizing follicular adenoma among FN category. Nevertheless a more extensive literature is necessary.

## Conclusion

The purpose of a thyroid FNAC is to identify nodules in a minimally invasive way that have a high ROM, thereby leading to appropriate surgery for concerning lesions and reducing the number of unnecessary surgical procedures for the other benign thyroid entities. The evaluation of patients with thyroid nodules undergoing FNAC should be based on a combination of clinical, radiological, and cytomorphological findings. Thyroid FNAC, however, remains challenging and there are many potential diagnostic pitfalls. Fortunately, there are several thyroid entities that exhibit very characteristic cytomorphologic features, making these lesions relatively easy to interpret based solely on their FNAC cytologic findings. For some of the more difficult diagnoses, the support of ancillary techniques is often necessary, whilst for some other very challenging lesions a conclusive diagnosis is impossible (Table 4). Awareness of these potential diagnostic pitfalls, combined with careful attention to cytologic and clinical features, along with judicious use of ancillary studies can help reduce errors, lead to more accurate FNAC interpretations and thus improve patient care.

## Data Availability

There is a local depositary system of cases including the details of o the cases used for the picture.
